# LMTK3 promotes tumorigenesis in bladder cancer via the ERK/MAPK pathway

**DOI:** 10.1002/2211-5463.12964

**Published:** 2020-09-16

**Authors:** Tao Jiang, Xinxing Lu, Feiya Yang, Mingshuai Wang, Hua Yang, Nianzeng Xing

**Affiliations:** ^1^ Department of Urology Beijing Chao‐Yang Hospital Capital Medical University Beijing China; ^2^ Department of Urology Affiliated Dalian Friendship Hospital of Dalian Medical University China; ^3^ Department of Urology National Cancer Center/National Clinical Research Center for Cancer/Cancer Hospital Chinese Academy of Medical Sciences and Peking Union Medical College Beijing China

**Keywords:** biomarker, bladder cancer, lemur tyrosine kinase‐3, weighted gene coexpression network analysis

## Abstract

Lemur tyrosine kinase 3 (LMTK3) is a key member of the serine–threonine tyrosine kinase family. It plays an important role in breast cancer tumorigenesis and progression. However, its biological role in bladder cancer remains elusive. In this study, we demonstrated that LMTK3 was overexpressed in bladder cancer and was positively correlated with bladder cancer malignancy. High LMTK3 expression predicted poor overall survival. Knockdown of LMTK3 in bladder cancer cells triggered cell‐cycle arrest at G2/M phase, suppressed cell growth, and induced cell apoptosis in bladder cancer cells. Furthermore, Transwell assays revealed that reduction of LMTK3 decreased cell migration by regulating the epithelial‐to‐mesenchymal transition pathway. Conversely, LKTM3 overexpression was shown to promote proliferation and migration of bladder cancer cells. We assessed phosphorylation of MEK and ERK1/2 in bladder cancer cells depleted of LMTK3 and demonstrated a reduced phosphorylation status compared with the control group. Using an MAPK signaling‐specific inhibitor, U0126, we could rescue the promotion of proliferation and viability in LMTK3‐overexpressing cells. In conclusion, we extend the status of LMTK3 as an oncogene in bladder cancer and provide evidence for its function via the activation of the ERK/MAPK pathway. Thus, targeting LMTK3 may hold potential as a diagnostic and prognostic biomarker and as a possible future treatment for bladder cancer.

## Graphical abstract

Here, we characterise LMTK3 as an oncogene in bladder cancer. LMTK3 was overexpressed in bladder cancer and this increased expression closely correlated with poor survival of bladder cancer patients. LMTK3 overexpression induced cell growth and migration whereas its depletion promoted apoptosis of bladder cancer cells. We found LMTK3 promoted tumorigenesis and progression by activating the ERK/MAPK pathway.

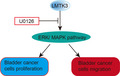

**Abbreviations**
EMT: epithelial‐to‐mesenchymal transition
ERK: extracellular regulated protein kinases
FBS: fetal bovine serum
GEO: Gene Expression Omnibus
GS: gene significance
LMTK3: lemur tyrosine kinase‐3
MAPK: mitogen‐activated protein kinase
ME: module eigengene
MTT: 3‐(4,5‐dimethylthiazol‐2‐yl)‐2,5‐diphenyl‐tetrazolium bromide
NC: normal control
PFA: paraformaldehyde
ROC: receiver operating characteristic
si‐LMTK3: LMTK3 target‐specific siRNA oligonucleotides
sLMTK3: soluble LMTK3
WGCNA: weighted gene coexpression network analysis

Bladder cancer is one of the most common malignant tumors worldwide. In China, the prevalence of bladder tumors ranks seventh among all malignant tumors in men [1], and the incidence is increasing year by year. The high morbidity and mortality of bladder cancer is a serious threat to human health. Early detection, early diagnosis and treatment, and delaying or even blocking the progression and metastasis of bladder cancer are the focus of clinical diagnosis and treatment. Therefore, exploring the pathogenesis of bladder cancer and seeking early diagnostic biomarkers and a therapeutic target have become the research hot spots in recent years.

Lemur tyrosine kinase‐3 (LMTK3) is an important member of the serine–threonine tyrosine kinase family. It can catalyze protein phosphorylation and directly or indirectly participate in a variety of cell signaling pathway transductions, such as estrogen receptor pathway, Wnt signaling pathway and forkhead box O3 pathway, among others. Previous research found that LMTK3 is highly expressed in many malignant tumors; its high expression is significantly correlated with the oncogenesis, progression and prognosis of various tumors [2, 3, 4]. Zhang *et al*. [5] found that LMTK3 is a novel biomarker for primary non‐small cell lung cancer, and high expression of LMTK3 predicts unfavorable clinical prognosis. The pathogenetic role of LMTK3 in breast cancer has been confirmed by several studies [6, 7, 8]. LMTK3 promotes breast cancer progression and decreased the sensitivity to doxorubicin treatment by decreased activity of ataxia‐telangiectasia mutated kinase [9]. Shi *et al*. [10] found that expression of preoperative soluble LMTK3 (sLMTK3) is an independent prognostic factor for patients with colorectal cancer compared with patients with low levels of sLMTK3, and patients with high sLMTK3 expression showed a poorer overall survival. However, the role of LMTK3 in bladder cancer has not been studied.

In this study, we revealed that LMTK3 was overexpressed in bladder cancer and was positively correlated with malignancy of bladder cancer. LMTK3 could be used as a diagnostic and prognostic biomarker in bladder cancer. Mechanistically, LMTK3 promoted bladder cancer cell proliferation and progression by activating the ERK/MAPK pathway.

## Materials and methods

### Bioinformatics analysis of *LMTK3* gene in bladder cancer

Gene expression matrix of bladder cancer dataset GSE13507 and clinical data were obtained from the Gene Expression Omnibus (GEO) database (http://www.ncbi.nlm.nih.gov/geo/). One hundred sixty‐six primary bladder cancer samples were used to perform weighted coexpression network analysis and identify clinically meaningful modules. We used weighted gene coexpression network analysis (WGCNA) r package to construct scale‐free gene coexpression networks. In brief, the similarity matrix based on the connection strength between each pair of genes was calculated. The expression of each module was summarized by module eigengene (ME), which can be considered a representative of the gene expression profiles in a module. Module membership is defined as the correlation between the ME and gene expression values. In addition, the gene significance (GS) was defined as mediated *P* value of each gene (GS = lgP) in the linear regression between gene expression and the clinical features. The module significance was defined as the average GS of all the genes involved in the module. Finally, clinically meaningful modules were identified. In addition, we performed receiver operating characteristic (ROC) curve to assess the efficacy of LMTK3 to distinguish between muscle‐invasive bladder cancer and non‐muscle invasive bladder cancer.

### Human bladder cancer tissue samples

Bladder cancer and adjacent normal tissue samples (*n* = 68) were obtained from patients after surgery at Dalian Municipal Friendship Hospital. Two pathologists performed the pathological diagnosis independently. The use of human bladder tissue samples was approved by the Institutional Review Board at the Dalian Municipal Friendship Hospital (Institutional Review Board number: 2019003). The study protocol and procedures conformed to the standards set by the latest revision of the Declaration of Helsinki. Informed written consents were obtained from all patients.

### Cell lines and cell culture

The human bladder cancer cell lines EJ (carcinoma, Cat. #CL‐0274) and UMUC3 (Cat. #TCHu217) were purchased from Cell Bank of Chinese Academy of Sciences (Shanghai, China). UMUC3 cells were cultured in Dulbecco’s modified Eagle’s medium (Gibco, Grand Island, NY, USA) supplemented with 10% fetal bovine serum (FBS) (Gibco), and EJ cells were cultured in RPMI‐1640 medium (Gibco, Beijing, China) containing 10% FBS. The cell lines were maintained in an incubator with a humidified atmosphere of 5% CO_2_ at 37 °C. The cell line was authenticated using short tandem repeat profiling and was mycoplasma negative as reported by routine laboratory examination.

### RNA isolation and quantitative real‐time PCR

Total RNA was isolated using HiPure Total RNA Mini Kit (Magen, Shanghai, China). NanoDrop was used to measure the quantity of isolated RNA. The reverse transcription reaction was performed according to the protocols on ReverTra Ace qPCR RT Kit (Toyobo, Shanghai, China). Real‐time quantitative PCR was then performed according to the protocols on SYBR Green PCR Master Mix (Bio‐Rad, Hercules, CA, USA). All primers of real‐time quantitative PCR were as follows: LMTK3: 5'‐ CAAGTGCTGTGGTTGTGTAATG‐3' (forward), 5'‐ CAGGCATCTTGTCGAGGATGG‐3' (reverse); GAPDH: 5'‐ GGAGCGAGATCCCTCCAAAAT‐3' (forward), 5'‐ GGCTGTTGTCATACTTCTCATGG‐3' (reverse). The relative expression of mRNA was normalized using GAPDH, and the data processing was performed using the 2−ΔΔCt method.

### Immunohistochemistry staining assays

Immunohistochemistry was performed as described previously [11]. In brief, after formalin fixation, paraffin embedding, section dewaxing, deparaffinization, hydration and antigen retrieval, the tissue samples were incubated with primary antibody. After a washing procedure, a broad‐spectrum secondary antibody was incubated. The sections were photographed with the Olympus BX53 biomicroscope. All staining was independently assessed by two pathologists. The immunoreactive scores were calculated by multiplying the staining intensity (negative = 0; weak = 1; moderate = 2; strong = 3) with the percentage of immunoreactive cells (0% = 0; 1–10% = 1; 11–50% = 2; 51–80% = 3; 81–100% = 4). The final scores were considered as negative (0; 0–1), weakly positive (1+; 2–4), moderately positive (2+; 6–8) and strongly positive (3+; 9–12).

### Knockdown and overexpression of *LMTK3* in the bladder cancer cells

LMTK3 target‐specific siRNA oligonucleotides (si‐LMTK3) were synthesized by View Solid (Beijing, China). The sense sequence of si‐LMTK3 is as follows: siRNA1: 5'‐GATGTCGGCTTCAAGGAATTT‐3'; siRNA2: 5'‐GCAAGTGCTGTGGTTGTGTAA‐3'; siRNA3: 5'‐GCTGCCGTTTCTGCTGATTAT‐3'. The target sequence of si‐control is 5ʹ‐UUCUCCGAACGUGUCACGUTT‐3ʹ. The plasmid of LMTK3 overexpression (pcDNA5/FRT/TO/LMTK3 vector) was synthesized by GenePharm biotech (Shanghai, China). When bladder cancer cell lines were grown to 50%, cells were transfected with siRNA or plasmid using Lipofectamine 2000 transfection reagent according to the manufacturer’s protocol. After 48‐h transfection, the cells were collected for subsequent experimental analysis.

### Immunofluorescence staining for bladder cancer cells

Coverslips were washed three times by cold PBS and fixed with 4% paraformaldehyde (PFA) for 30 min. Then the cells were treated by 0.1% Triton X‐100 and blocked in goat serum for 30 min, incubated with primary antibody (Table S1) at room temperature for 2 h, washing with PBS and incubating with Cy3‐labeled or fluorescence isothiocyanate‐labeled secondary antibody for 1 h. Nuclei were labeled with DAPI (2 μg·mL^−1^). Immunofluorescence staining was analyzed using a fluorescence microscope (cat. no. IX73; Olympus, Tokyo, Japan).

### Flow cytometry analysis for cell cycle and apoptosis

For cell cycle, bladder cancer cells were washed with PBS three times, then resuspending cells with 1 × DNA Staining Solution containing permeabilization solution and propidium iodide (Cat. #CCS012; Multi Sciences, Hangzhou, China) for 30 min at 37 °C in the dark. Flow cytometry (cat. no. FC500; Beckman Coulter,Brea, CA, USA) was conducted to assess the cell cycle. For cell apoptosis, according to the manufacturer's instructions, cell apoptosis analysis was performed using the Annexin V‐fluorescence isothiocyanate/propidium iodide apoptosis detection kit (Cat. #558547; BD Biosciences, San Jose, CA, USA) by flow cytometry.

### Cell proliferation assay

Cell proliferation was examined using 3‐(4,5‐dimethylthiazol‐2‐yl)‐2,5‐diphenyl‐tetrazolium bromide (MTT) assay and cell colony formation assay. For MTT assay, after transfection for 48 h, bladder cancer cells were plated in 96‐well plates (3000 cells/well) to grow for another 5 days. Then 24 h per interval, 20 μL MTT was added into each well and incubated for 4 h at 37 °C. Then the medium was removed, 200 μL DMSO was added and absorbance value at 490 nm (*A*_490 nm_) was measured by a microplate reader (cat. no. SpectraMax M2; Molecular Devices, Sunnyvale, CA, USA). For cell colony formation assay, bladder cancer cells were plated in six‐well plates (1000 cells/well) and grew into colonies for approximately 15 days. Colonies emerged and were fixed by 4% PFA for 30 min and stained with 0.1% crystal violet for observation and photographing.

### Cell migration and invasion assays

Transwell chambers (Corning, Inc., NY, USA) were used for cell migration and invasion assays. For cell migration assays, a total of 3 × 10^4^ cells were cultured with serum‐free medium in each upper chamber (Corning, Inc.). Meanwhile, the lower chamber was filled with medium supplemented with 10% FBS. After culturing for 24 h at 37 °C, the cells of the upper chamber were removed using cotton swabs. Then the lower side of the chamber was fixed by 4% PFA for 30 min and stained with 0.1% crystal violet for observation and photographing; migrated cell number was counted and statistically analyzed. For cell invasion assay, first adding ECM Matrix gel solution (Sigma‐Aldrich, St. Louis, MO, USA) in Transwell chambers and then solidifying at 37 °C, the subsequent incubation, staining and observation procedures were identical to the migration assays.

### Western blot analysis

The radioimmunoprecipitation assay buffer pierce supplemented with protease inhibitor and phosphatase inhibitor (Sigma‐Aldrich) was used to isolate bladder cancer cells protein. After being boiled, samples were loaded in SDS/PAGE and transferred to poly(vinylidene difluoride) membranes (Millipore, Billerica, MA, USA). Poly(vinylidene difluoride) membranes were blocked in 5% nonfat milk, then incubated with primary antibodies with gentle shaking at 4 °C overnight and subsequently incubated with secondary antibodies. The immunoreactive bands were visualized by Molecular Imager ChemiDoc XRS + Imaging system (Bio‐Rad, USA). The primary antibodies and secondary antibodies were described in Table S1.

### Rescue experiments using MAPK inhibitor

Before LMTK3 plasmid transfection, bladder cancer cells were pretreated using MAPK inhibitor U0126 (Sigma, USA) at 10 μm for 2 h to rule the ERK/MAPK signaling. The normal control (NC) group was pretreated with 0.1% DMSO. After 48‐h transfection of LMTK3 plasmid, the cells were collected for subsequent experimental analysis.

### Statistical analysis

r software 3.5.0 (Auckland, New Zealand) was performed for all analyses. The significance of differences between subgroups was analyzed by two independent sample *t*‐tests, and χ^2^ test was performed to analyze the correlation between LMTK3 expression and clinicopathological characteristics. Kaplan–Meier survival curves were built to analyze survival differences between the high‐expression group and low‐expression group and compared by log rank test. Univariate and multivariate Cox proportional hazard models were performed to estimate the hazard ratios of prognostic factors; Wald test was applied to compare whether an independent variable has a statistically significant relationship with a dependent variable. Statistical significance was set at probability values of *P* < 0.05.

### Results

### Bioinformatics analysis of *LMTK3* gene in bladder cancer

**Fig. 1:**
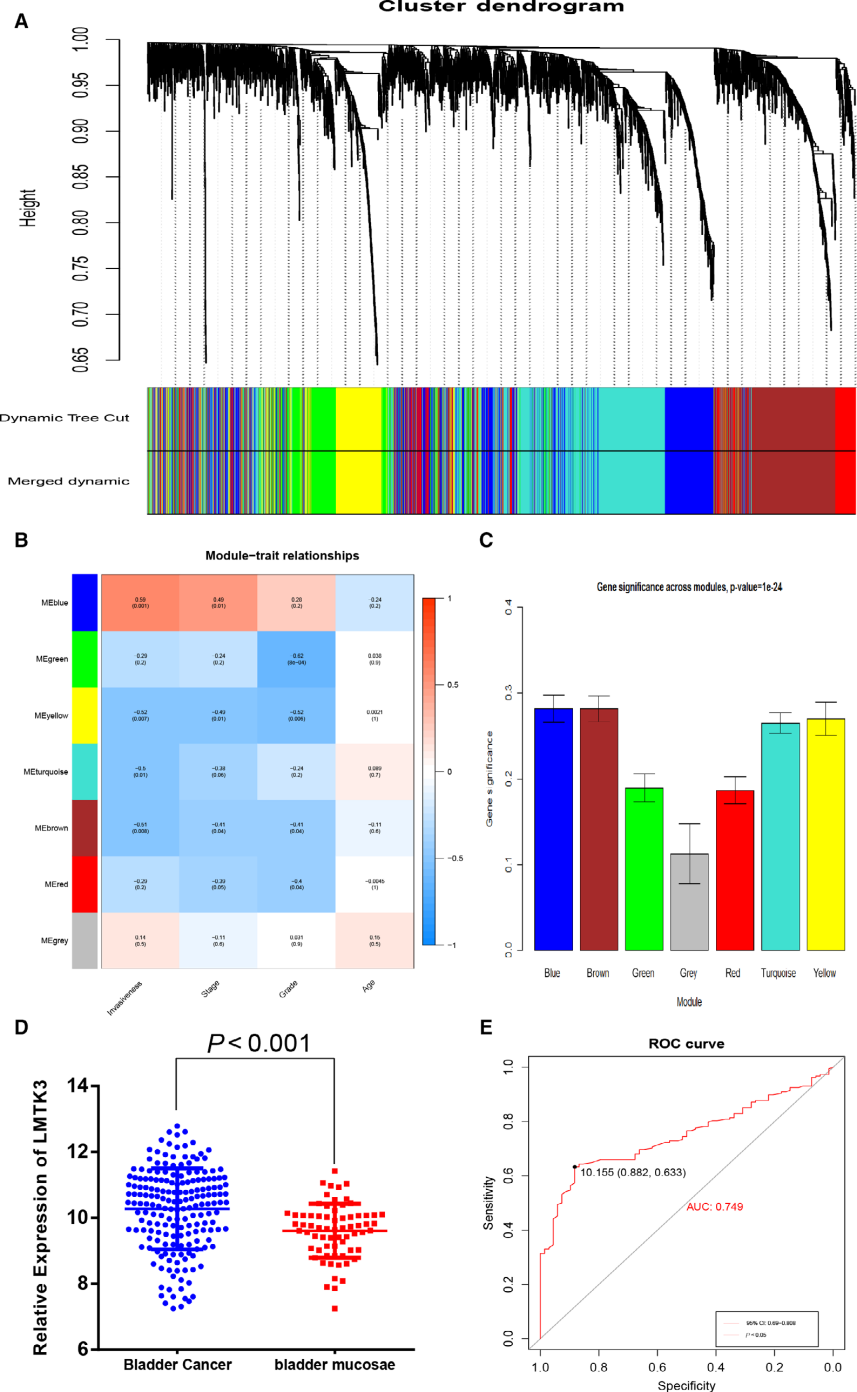
Bioinformatics analysis of LMTK3 in bladder cancer. (A) Dendrogram of all differentially expressed genes clustered based on a dissimilarity measure (1‐Topological Overlap Measure). (B) Heatmap of the correlation between MEs and different clinical information of bladder cancer (invasiveness, stage, grade and age). (C) Distribution of the average gene significance and errors in the modules associated with the stage of bladder cancer. (D) The comparison of LMTK3 expression in 188 bladder cancer tumor tissues and 68 paracarcinomatous tissues. Comparison was performed with Student’s *t*‐test. Data are presented as the mean ± SD. (E) Diagnostic value analysis of LMTK3 in bladder cancer with excellent specificity and sensitivity.

**Fig. 2:**
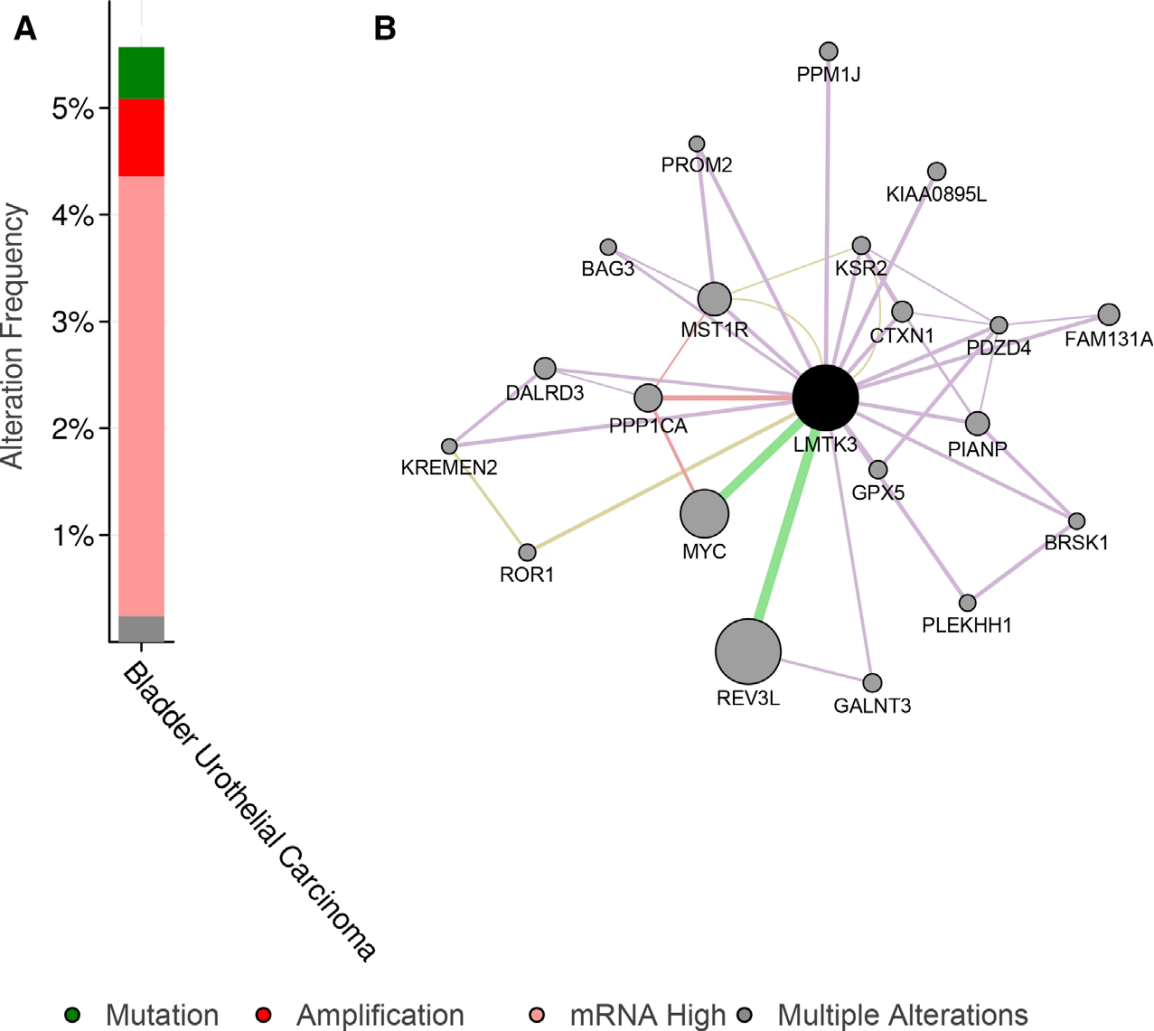
The altered types and the regulatory network of LMTK3. (A) The main altered types of LMTK3 were amplification and mRNA up‐regulation in patients with bladder cancer. (B) The generated network by GeneMANIA showed that there were numerous interactions and coexpression between *LMTK3* and other cell‐cycle highly relevant genes.

Expression matrix of the GSE13507 dataset was used to perform WGCNA; the most clinically significant module was identified according to the relevance between each module and bladder cancer progression. Eventually blue module was identified as the most significant module (Fig. 1A–C, *P* < 0.05). LMTK3 indicated a significant correlation with the blue module (*R* = 0.59). ROC curve was used to assess the capacity of LMTK3 to distinguish between bladder cancer and adjacent normal tissues, and the area under the curve value for LMTK3 was 0.749 (Fig. 1E). LMTK3 also showed significantly higher expression level in bladder cancer tissues as compared with adjacent normal tissues (Fig. 1D, *P* < 0.05). Then the cBioPortal database showed that the main altered types of LMTK3 were amplification and mRNA up‐regulation in patients with bladder cancer (Fig. 2A). The generated network of GeneMANIA [12] (http://genemania.org/) showed that there were numerous interactions and coexpression between *LMTK3* and other cell‐cycle highly relevant genes (Fig. 2B).

### *LMTK3* was overexpressed in bladder cancer tissues

**Fig. 3:**
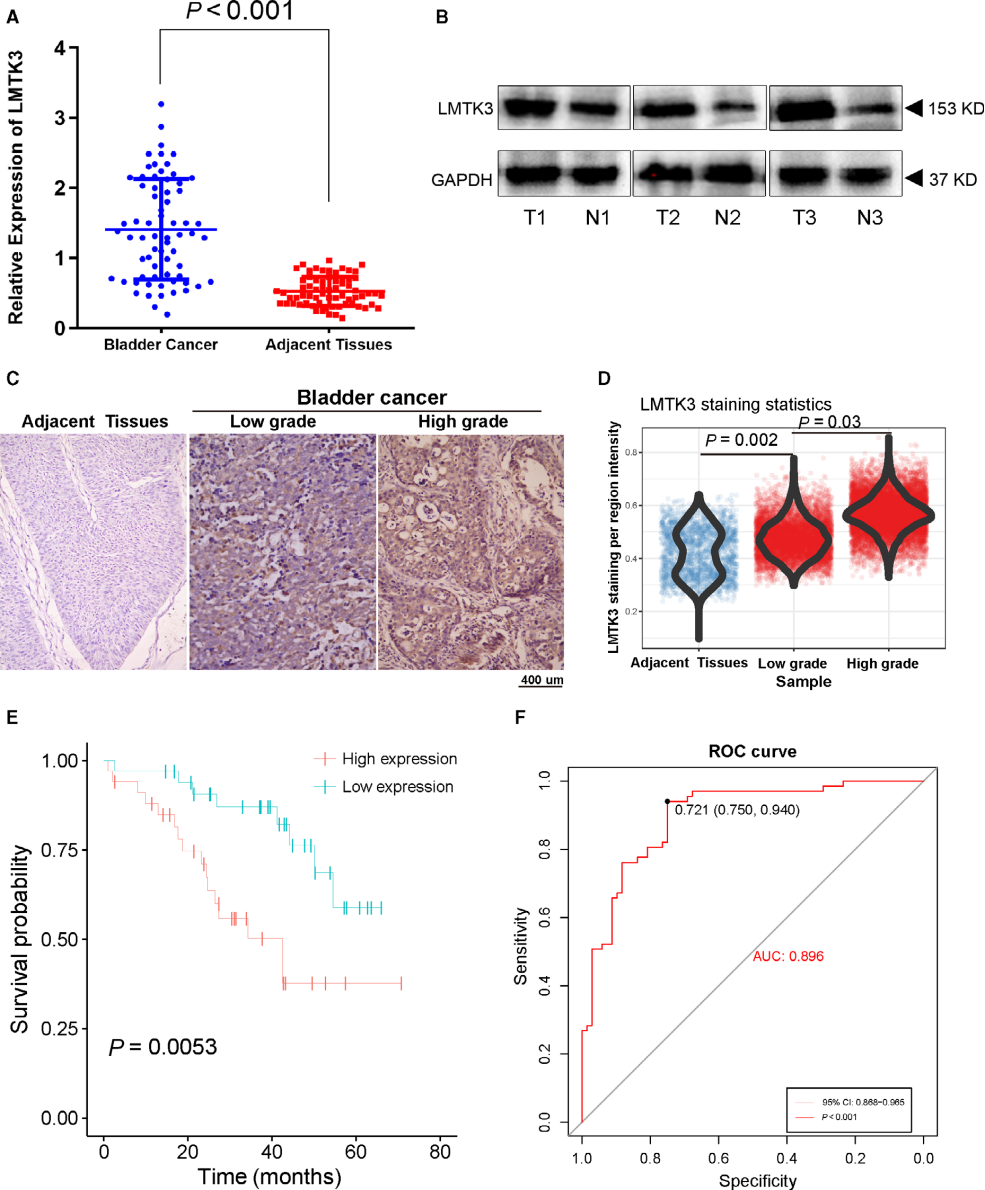
LMTK3 is a diagnostic and prognostic factor. LMTK3 expression and prognostic value were analyzed based on 68 bladder cancer tissues and matched paracancerous tissues from the Department of Urology in our hospital. (A) LMTK3 mRNA expression is up‐regulated in 68 bladder cancer tissues compared with matched paracancerous tissues. (B) Western blot results show that LMTK3 protein expression is up‐regulated in three bladder cancer tissues and paired paracancerous tissues. (C, D) Immunohistochemical staining results show the protein expression of LMTK3 in 68 bladder cancer tissues and paired paracancerous tissues. (E) Kaplan–Meier survival analysis reveals that LMTK3 expression predicts a poor prognosis in 68 patients with bladder cancer. (F) ROC curves analysis of LMTK3. Data are presented as the mean ± SD; statistical analysis was performed, and comparison was performed with Student’s *t*‐test. Scale bar: 400 μm.

Sixty‐eight bladder cancer tissues and paracancerous tissues were used to explore LMTK3 expression in bladder cancer. The result of real‐time quantitative PCR and western blot analyses showed that LMTK3 was significantly overexpressed in bladder cancer tissues as compared with paracancerous tissues (Fig. 3A,B). Accordingly, the result of immunohistochemistry staining indicated that LMTK3 protein expression was abnormally up‐regulated in the bladder cancer tissues as compared with paracancerous bladder tissues. Moreover, the LMTK3 expression was positively correlated with tumor grade (Fig. 3C).

### Correlation of LMTK3 expression with clinical characteristics in bladder cancer

To explore the correlation between LMTK3 and patients’ clinicopathological information, we collected clinical information, including age, sex, grade and stage. Tumor grade classification was based on the World Health Organization classification system of tumors of the urinary system, and stage classification was carried out according to the 2010 American Joint Committee on Cancer TNM classification [13, 14]. All of the patients were divided into the high‐expression group and low‐expression group based on the median value of LMTK3 expression. The LMTK3 expression groups and clinicopathological data were listed in Table 1. LMTK3 expression status was not correlated with age (*P* = 0.649) or sex (*P* = 0.969); however, stage (*P* < 0.001) and grade (*P* < 0.05) showed a significant relevance with LMTK3 expression.

**Table 1:** Association between the expression of LMTK3 and clinicopathological characteristics.

Variable	Total patients	LKTK3 expression		
		Low	High	*P*
Age, mean ± SD (years)	59.5 ± 11.2	58.9 ± 10.2	59.7 ± 9.5	0.738
Age, n (%)				0.649
<65 years	28 (41.2)	18 (64.3)	10 (35.7)	
≥65 years	40 (58.8)	29 (72.5)	11 (27.5)	
Sex, n (%)				0.969
Male	47 (69.1)	22 (46.8)	25 (53.2)	
Female	21 (30.9)	9 (42.9)	12 (57.1)	
Stage, n (%)				<0.001
II	24 (11.0)	19 (79.2)	5 (20.8)	
III	38 (23.0)	13 (34.2)	25 (65.8)	
IV	6 (15.3)	1 (16.7)	5 (83.3)	
Grade, n (%)				0.039
Low	23 (33.8)	15 (65.2)	8 (34.8)	
High	45 (66.2)	16 (35.6)	29 (64.4)	

### LMTK3 was an independent diagnostic and prognostic factor in bladder cancer

The diagnostic value of LMTK3 was evaluated using the ROC curve in bladder cancer. The result showed the area under the curve of the ROC curve was 0.896 (95% confidence interval, 0.812–0.913; *P* < 0.001) with 75.0% sensitivity, 94.0% specificity and a diagnostic threshold value of 0.721 (Fig. 3E). Then choosing median expression of LMTK3 as cutoff value, we assigned patients to the high‐expression group and low‐expression group. Patients in the high‐expression group showed poorer prognoses, whereas patients in the low‐expression group had more favorable prognoses (*P* = 0.0053; Fig. 3D). Because stage and grade were risk factors of bladder cancer, we used Cox regression analyses to explore whether LMTK3 was an independent prognostic factor. The result showed that, even adjusted by stage and other covariates, LMTK3 remained an independent prognostic factor (Table 2).

**Table 2:** Cox regression analysis for patients with bladder cancer. CI, confidence interval; HR, hazard ratio.

Variable	Univariable Cox regression			Variable	Multivariable Cox regression		
	HR	95% CI	*P* value		HR	95% CI	*P* value
LMTK3	2.547	1.335–5.991	0.005	LMTK3	1.441	1.191–2.882	0.023
TNM stage	4.434	2.162–9.660	<0.001	TNM stage	2.834	1.862–6.660	0.003
Grade	3.443	1.831–5.650	<0.001	Grade	2.443	1.431–5.650	0.005

### Down‐regulation of LMTK3 inhibits proliferation and migration of bladder cancer cells

**Fig. 4:**
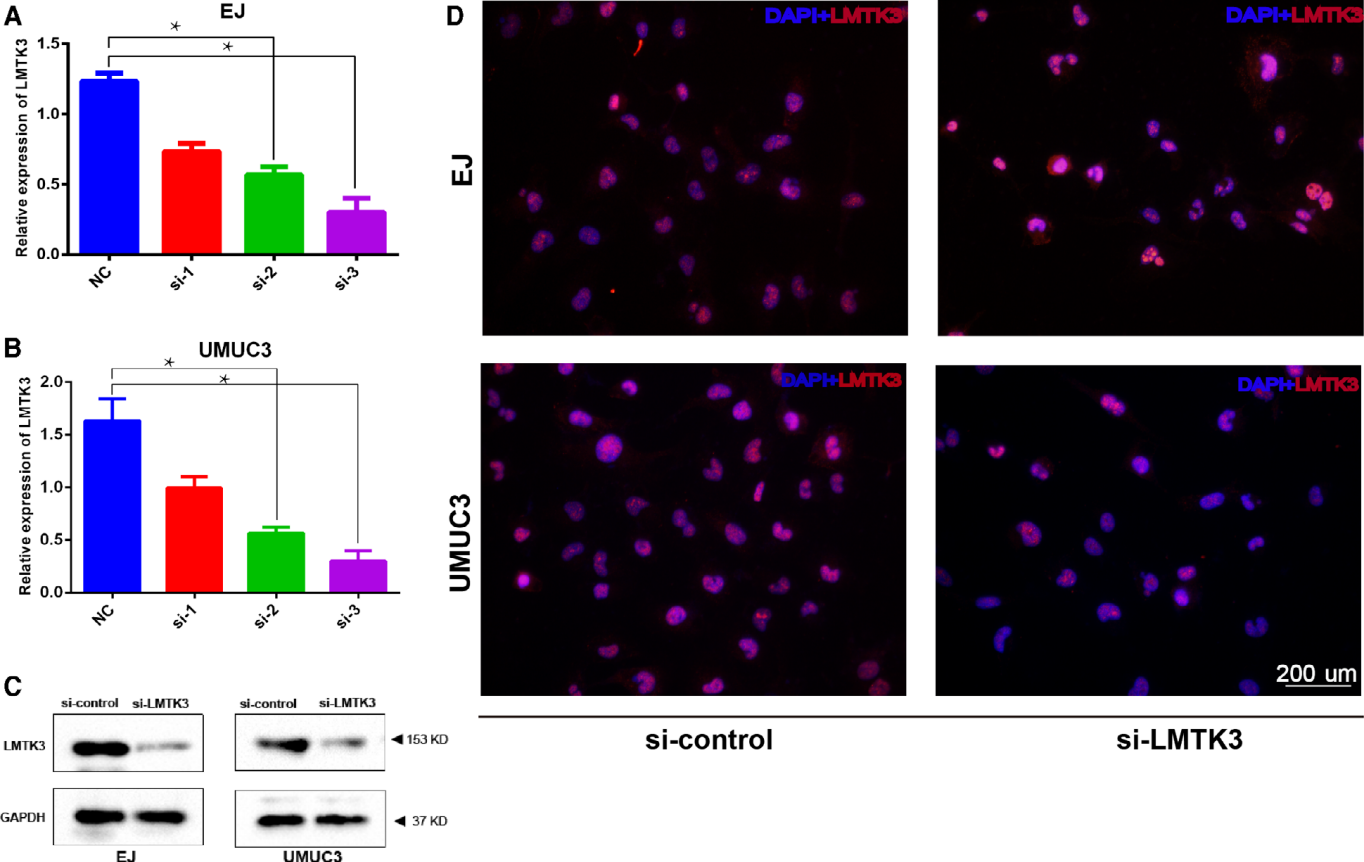
Knockdown *LMTK3* gene with siRNA. (A, B) Real‐time quantitative PCR analysis of the knockdown efficiency of LMTK3 siRNA in EJ and UMUC3 cells. (C) Western blot assay revealed a significantly decreased protein abundance of LMTK3 by the si‐LMTK3 knockdown as compared with si‐control treatment. (D) Representative immunofluorescence staining of LMTK3 (red) in the si‐LMTK3 and si‐control groups. Data represent the mean ± SD of three separate experiments. Differences between two groups were compared using Student’s *t*‐test. *P < 0.01. Scale bar: 200 μm.

**Fig. 5:**
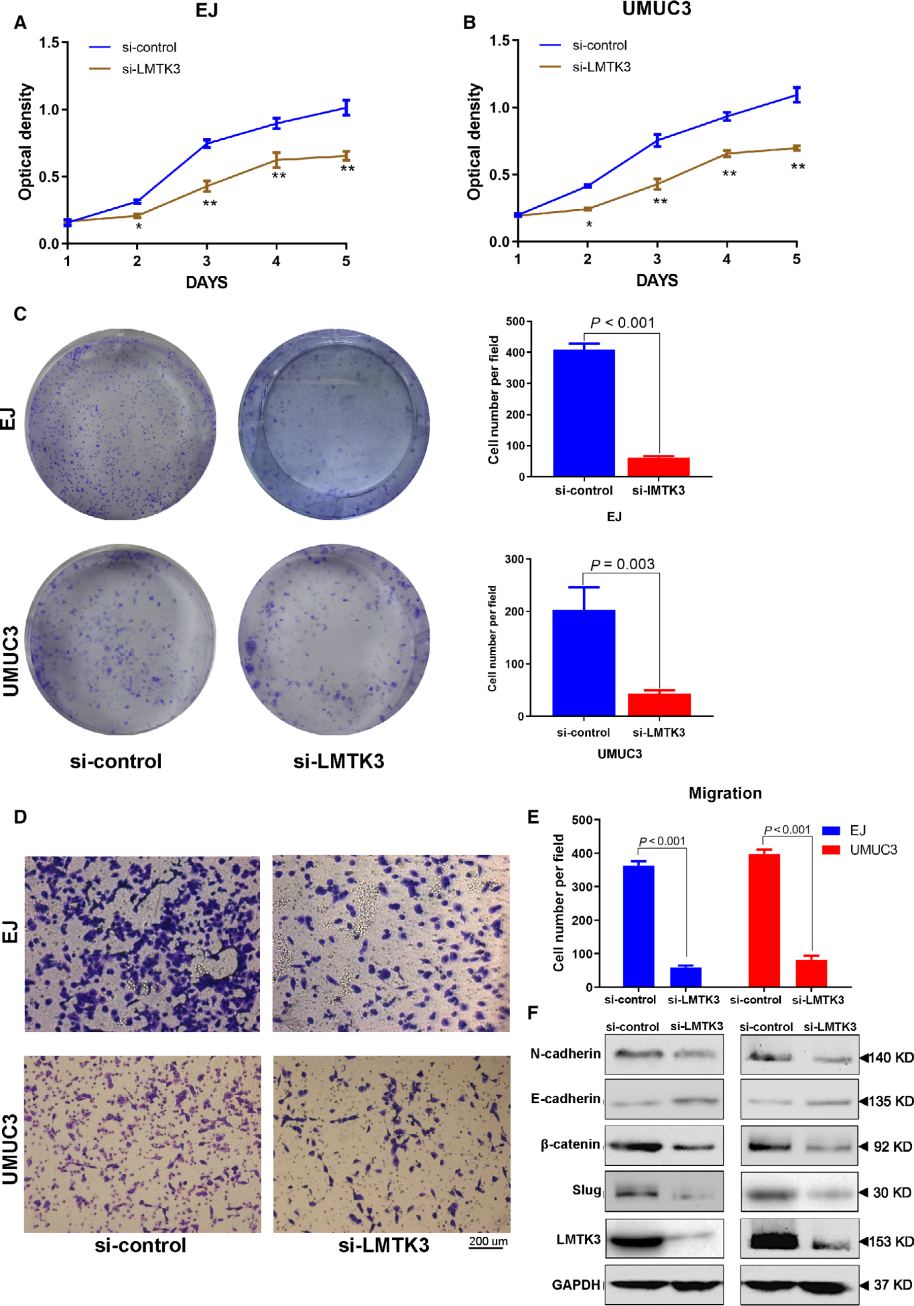
Down‐regulation of LMTK3 represses bladder cancer cell proliferation and migration. (A, B) MTT assay was used to detect the viability of bladder cancer cells in the si‐LMTK3 and si‐control groups. (C) Cell colony formation assay detects alteration of cell survival for EJ and UMUC3 in the si‐LMTK3 and si‐control groups. Clone number in each well was counted and statistically analyzed in the cell colony formation assay. (D) Cell migration analysis of the si‐LMTK3 and si‐control groups in bladder cancer cells. (E) Statistical analysis of cell migration. (F) Western blot analysis of proteins involved in the EMT pathway. Data represent the mean ± SD of three separate experiments. Differences between two groups were compared using Student’s *t*‐test. *P < 0.05, **P < 0.001. Scale bar: 200 μm.

To explore the biological effects of LMTK3 in bladder cancer cells, we transfected three LMTK3‐siRNA into EJ and UMUC3 cells. The knockdown efficiency was analyzed using real‐time quantitative PCR, western blot and immunofluorescence staining. All of the results showed that LMTK3 was significantly down‐regulated at both transcriptional and translational levels after LMTK3‐siRNA transfected into the bladder cancer cells (Fig. 4). Then MTT assay and cell colony formation assay were performed to elucidate the potential effect of LMTK3 promoting the proliferation and viability of bladder cancer cells. The results of MTT assay identified that LMTK3 knockdown obviously inhibited cell proliferation (Fig. 5A,B). Cell colony formation assay revealed that cell colony‐forming efficiency was reduced with LMTK3 knockdown (Fig. 5C). All of the results showed that LMTK3 knockdown suppresses cell growth. We further investigated whether LMTK3 regulated migration of bladder cancer cells using Transwell migration assay. The results showed that the numbers of cell migration were obviously reduced with the knockdown of LMTK3 in bladder cancer cells (Fig. 5D), and the statistical analysis was consistent with the result (Fig. 5E). The epithelial‐to‐mesenchymal transition (EMT) pathway plays a pivotal role in cancer migration and invasion [15]. The western blot results revealed that the epithelial key marker E‐cadherin was elevated; in contrast with the mesenchymal marker N‐cadherin, Slug and β‐catenin were degraded with LMTK3 knockdown (Fig. 5F). These data indicated that LMTK3 triggered the EMT process to impel cell migration and invasion.

### LMTK3 knockdown leads to G2/M cell‐cycle arrest and apoptosis in bladder cancer cells

**Fig. 6:**
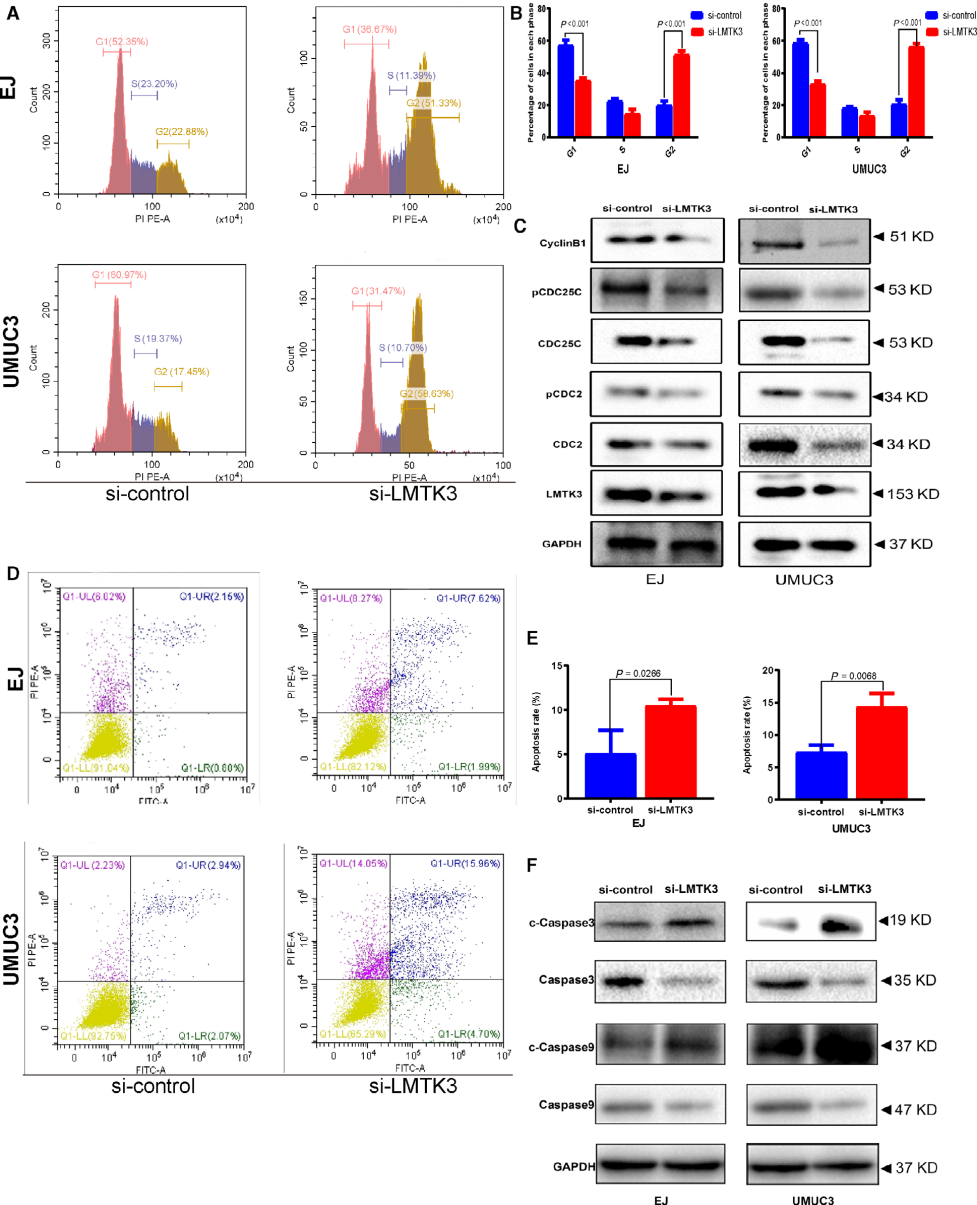
Knockdown of LMTK3 induces cell apoptosis and cell‐cycle arrest at G2/M phase. (A, B) Cell‐cycle analysis of si‐LMTK3 and si‐control groups in bladder cancer cells. (C) Western blot analysis showed the variety of proteins involved in the G2/M cell cycle. (D, E) Flow cytometry analysis of cell apoptosis of si‐LMTK3 and si‐control groups in bladder cancer cells. (F) Western blot analysis showed the variety of proteins involved in the cell apoptosis. All values are presented as the mean ± SD from three independent research results; comparison was performed with Student’s *t*‐test.

To explore the effect of LMTK3 in regulating the cell‐cycle progression of bladder cancer cells, we performed flow cytometric analysis on UMUC3 and EJ cells. After bladder cancer cells were transfected with LMTK3‐siRNA for 48 h, an obvious accumulation of cells in the G2/M phase was shown and accompanied a reduction of cells in G0/G1 phase (Fig. 6A,B). Moreover, after bladder cancer cells transfected with LMTK3‐siRNA, western blot analysis revealed the key proteins associated with G2/M phase [16, 17] were down‐regulated, including cyclin B1, Cdc25c, Cdc2, as well as their phosphorylated counterparts (Fig. 6C). We also used the Annexin V apoptosis assay to investigate whether silencing LMTK3 expression could induce apoptosis in bladder cancer cells. The result revealed that there was a significant increase in the apoptotic population in UMUC3 and EJ cells transfected with LMTK3‐siRNA (Fig. 6D,E). The expressions of key proteins, such as cleaved caspase‐3 and cleaved caspase‐9, were reduced (Fig. 6F). These results further indicated the oncogenic role of LMTK3 in bladder cancer cells through the regulation of the G2/M cell cycle and apoptosis.

### Overexpression of LMTK3 promotes proliferation and migration of bladder cancer cells

**Fig. 7:**
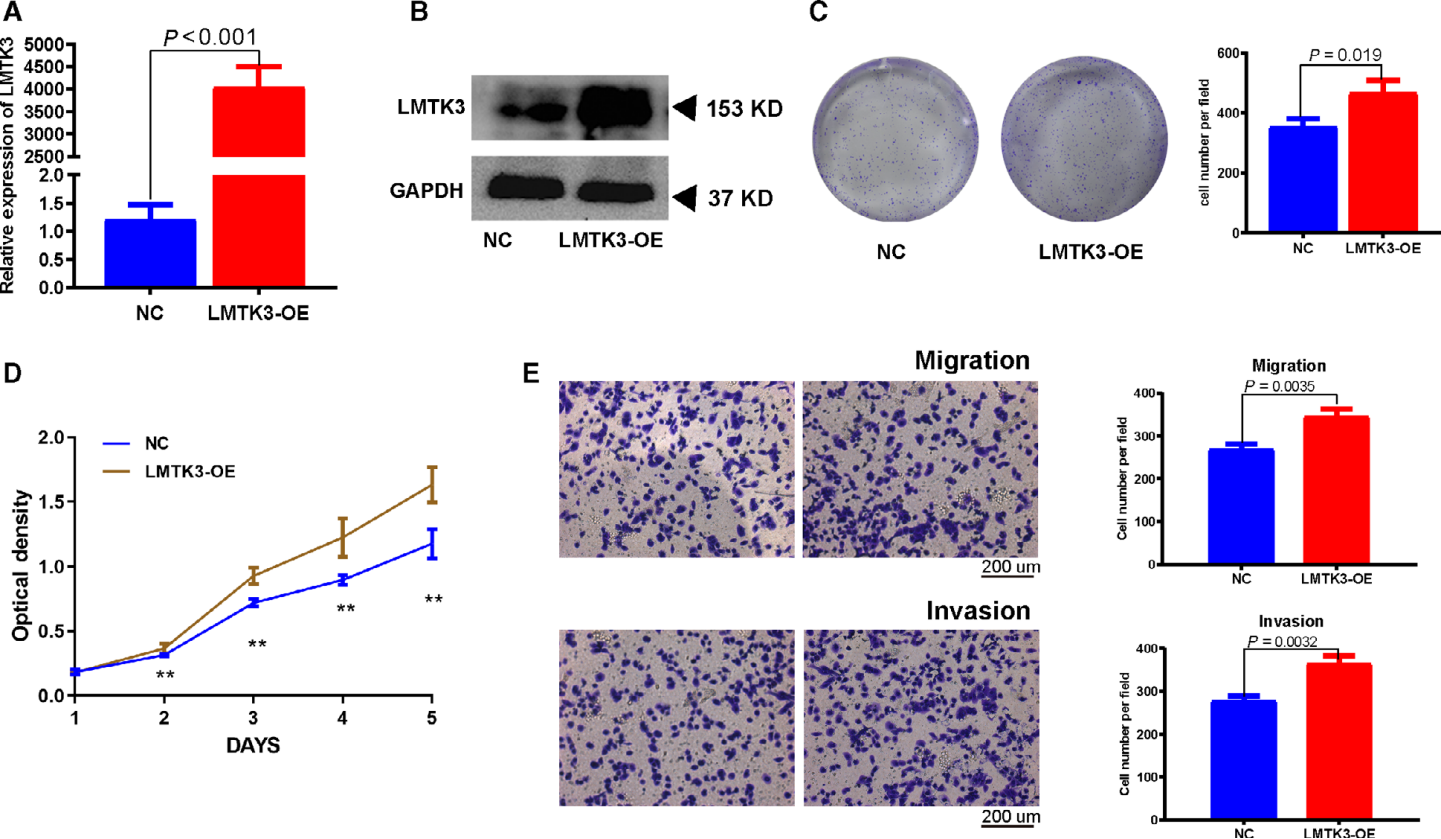
Overexpression of LMTK3 promotes proliferation and migration of bladder cancer cells. (A, B) Real‐time quantitative PCR and western blot analyses verified the overexpressed efficiency of LMTK3. (C) Cell colony formation of NC and LMTK3‐OE groups in EJ cells. (D) MTT assay of NC and LMTK3‐OE groups in EJ cells. (E) Transwell migration assay of NC and LMTK3‐OE groups in EJ cells. Statistical analysis was performed. All of the values shown are mean ± SD of triplicate measurements; comparison was performed with Student’s *t*‐test. Scale bars: 200 μm. GAPDH, glyceraldehyde‐3 phosphate dehydrogenase; OE, overexpression; ** <0.01.

To assess the biological effects of LMTK3 overexpression in bladder cancer cells, we transfected LMTK3 plasmid into EJ cells. The overexpression efficiency was validated by real‐time quantitative PCR and western blot assay. The results showed that the LMTK3 was significantly up‐regulated at both transcriptional and translational levels after LMTK3 plasmid transfected into the EJ cells (Fig. 7A,B). The results of cell colony formation assay showed that LMTK3 overexpression obviously promoted cell colony‐forming efficiency and cell proliferation (Fig. 7C). The MTT assay revealed that LMTK3 overexpression promoted EJ cells proliferation activity (Fig. 7D). The migration efficiency was increased with LMTK3 overexpression (Fig. 7E).

### LMTK3 promotes bladder cancer cells proliferation and migration through the ERK/MAPK signaling pathway

**Fig. 8:**
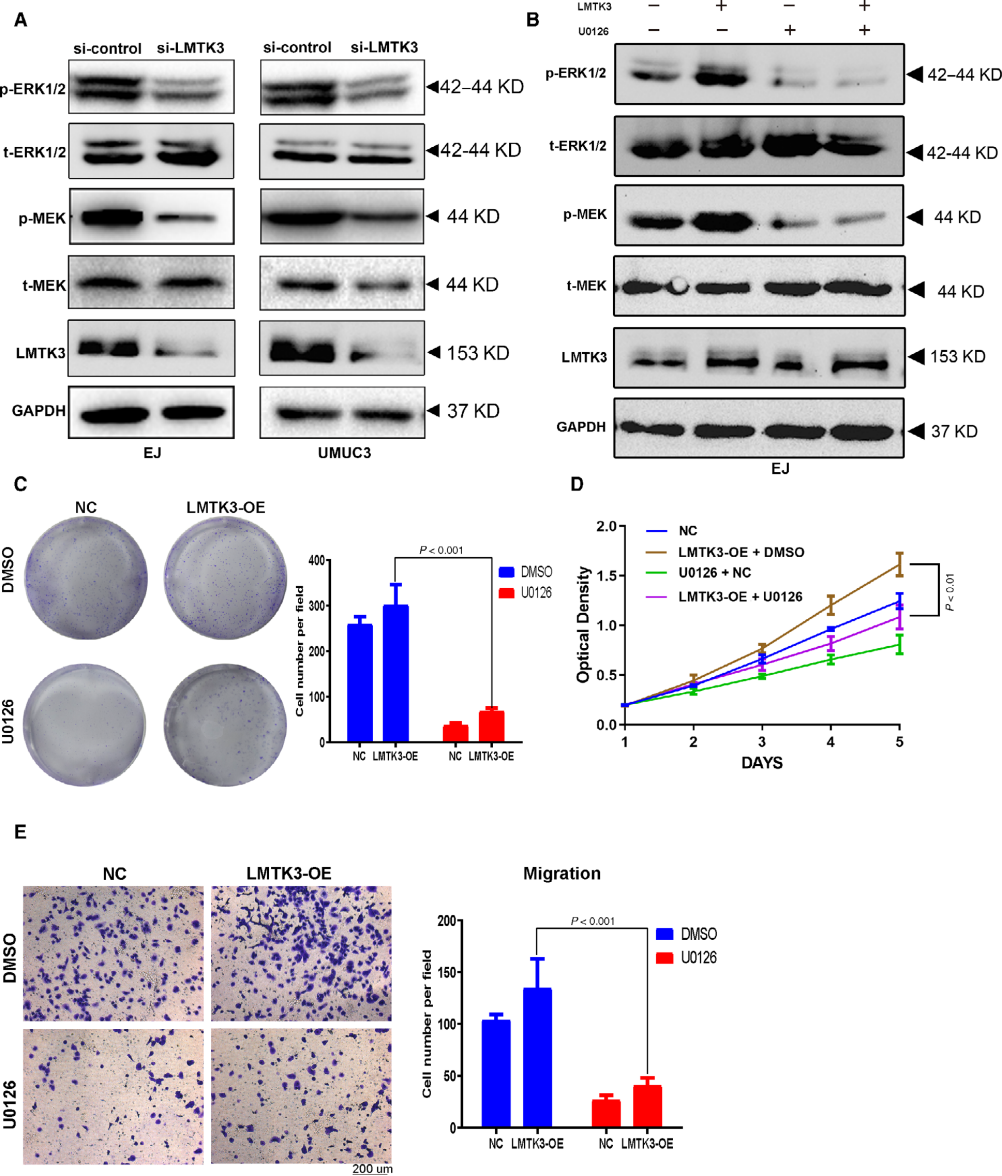
LMTK3 promotes bladder cancer cells proliferation and migration through the ERK/MAPK signaling pathway. (A) The protein levels of LMTK3 and ERK/MAPK markers in si‐LMTK3 and si‐control bladder cancer cells were estimated by western blot. (B) The protein levels of LMTK3 and ERK/MAPK signaling pathway‐related markers in LMTK3‐overexpression EJ cells [treated with MAPK inhibitor U0126 (10 μm)] were assessed by western blot. (C–E) Rescue experiments of LMTK3 overexpression by using MAPK inhibitor U0126 (10 μm): cell colony formation, MTT and Transwell migration assay. Data represent the mean ± SD of three separate experiments; comparison was performed with one‐way ANOVA followed by Tukey’s *post hoc* test. Scale bar: 200 μm.

The activation of the ERK/MAPK signaling pathway has a pivotal role in oncogenesis and progression of bladder cancer [18, 19, 20, 21]. Key members of the ERK/MAPK signaling pathway include ERK1/2 and MEK. Previous research suggested that LMTK3 may be involved in tumor progression via the activation of the ERK/MAPK pathway [3]. Our western blot results showed the phosphorylation of MEK and ERK1/2 in the si‐LMTK3‐treated group was decreased as compared with the si‐control group (Fig. 8A). To further identify whether LMTK3 regulated bladder cancer cells proliferation and migration through the ERK/MAPK pathway, we used the MAPK signaling‐specific inhibitor U0126 to rule the ERK/MAPK signaling function in EJ cells. The cell colony formation assay showed that U0126 could significantly rescue the promotion of proliferation and viability in LMTK3 overexpression EJ cells (Fig. 8C); the MTT and Transwell migration assay also revealed identical results (Fig. 8D,E). Furthermore, we also observed that changes of ERK/MAPK markers expression (ERK1/2, MEK) reasoned by LMTK3 overexpression can be recovered by U0126 (Fig. 8B). Collectively, these results indicated that LMTK3 regulated bladder cancer cells proliferation and migration through the ERK/MAPK signaling pathway.

## Discussion

Currently, with the development of genomic sequencing, various public bioinformatics databases are available to researchers. It becomes possible to analyze the biological functions of genes in tumors using bioinformatics big data. WGCNA is a popular tool for multisample complex data analysis. Based on WGCNA, all of the genes are divided into multiple modules by calculating the correlation coefficient between genes. Finally, through the correlation analysis between these modules and sample phenotypes, a regulatory network among genes in the module is identified and key genes are selected out. In this study, we found by WGCNA analysis that LMTK3 was a hub gene in bladder cancer. It was associated with tumorigenesis and progression of bladder cancer. Then we clarified the clinical significance and oncogenic role of LMTK3 in bladder cancer. Further studies clarified that LMTK3 may regulate these biological functions by the ERK/MAPK signaling pathway.

Emerging evidence showed that LMTK3 played a pivotal role in the tumorigenesis and progression of most cancers [2, 3, 4]. In 2011, Giamas *et al*. [8] identified LMTK3 as a therapeutic target in breast cancer. Their team used the siRNA screen library to identify kinases whose silencing alters the estrogen response in breast cancer. They found that LMTK3 was one of the potent regulators. Further, they found that LMTK3 increased binding of forkhead box O3 to the ESR1 promoter by decreasing the activity of the phosphorylation of AKT and protein kinase C. Moreover, LMTK3 phosphorylated estrogen receptor α, protecting it from proteasomal degradation *in vitro*. Then they used breast cancer cases to explore the relationship of clinical pathological features and LMTK3. The results showed higher LMTK3 expression was correlated with progression and poor prognosis in breast cancer [22]. Interestingly, LMTK3 also enhanced resistance to endocrine therapy in breast cancer by reducing autophagy [23] and confers chemoresistance in breast cancer by the formation of γH2AX foci [9]. It has also been reported that *LMTK3* was an oncogene and a significant prognostic marker in colorectal cancer, lung cancer, gastric cancer, gastrointestinal stromal tumor and melanoma [24, 25, 26, 27, 28]. Our study showed that LMTK3 was up‐regulated in bladder cancer compared with paracancerous tissue; then the clinic data analysis indicated that high LMTK3 expression status was associated with tumor grade and stage. The Kaplan–Meier survival analysis showed that higher LMTK3 expression predicted a poorer prognosis, and multivariate Cox regression analyses exhibited that LMTK3 was an independent prognostic factor, so LMTK3 could also be a potential prognostic biomarker and therapeutic target. Then we used bladder cancer cells to explore the biological role of LMTK3. We found that the capability of bladder cancer cells proliferation and migration was decreased when the *LMTK3* gene was knocked down. These results further identified that LMTK3 played an oncogene role in bladder cancer. Previous studies reported that the ERK/MAPK signaling pathway plays an important function in bladder cancer [3, 29, 30, 31, 32]. LMTK3 may process biological function through the ERK/MAPK pathway in various tumors [3, 33]. Hence we focused on MAPK/ERK signaling to further explore the potential molecular mechanism of LMTK3 in bladder cancer. Our study showed the phosphorylation of MEK and ERK1/2 in the si‐LMTK3‐treated group was decreased compared with the si‐control group in bladder cancer cells. The MAPK signaling‐specific inhibitor U0126 could significantly rescue the promotion of proliferation and migration in LMTK3 overexpression EJ cells. Therefore, these results indicated that LMTK3 regulated cell proliferation and migration through the ERK/MAPK signaling pathway.

However, there are some limitations in this study. Our findings should be validated using *in vivo* gain‐ and loss‐of function studies. In addition, the molecular mechanisms of LMTK3 regulating the ERK/MAPK signaling pathway on the progression of bladder cancer should be elucidated. In conclusion, our research indicates for the first time that *LMTK3* is an oncogene in bladder cancer. It is a potential independent prognostic factor. *In vitro*, down‐regulation of LMTK3 inhibits bladder cancer cells proliferation and migration, and induces cell‐cycle arrest and apoptosis. In biological mechanisms, LMTK3 may process this effect by the ERK/MAPK signaling pathway. It could be a potential biomarker for the diagnosis and treatment of bladder cancer.

## Conflict of interest

The authors declare no conflict of interest.

## Author contributions

TJ, XL and FY conceived and designed the study. MW and HY performed the analysis procedures. TJ, XL and NX analyzed the results. TJ, MW and HY contributed analysis tools. TJ, XL and FY performed biological experiments. TJ, XL and NX contributed to the writing of the manuscript. All authors reviewed the manuscript.

## Supporting information


**Table S1.** List of antibodies.

## Data Availability

Data were deposited in and are accessible through NCBI's GEO Series accession number GSE13507. The data are available from the corresponding author upon reasonable request.
