# Corrigendum to: LMTK3 promotes tumorigenesis in bladder cancer via the ERK/MAPK pathway

**DOI:** 10.1002/2211-5463.13116

**Published:** 2021-03-02

The affiliation of the corresponding author of the paper by Jiang *et al*. [1] was incomplete. The full affiliation is provided here:

**Nianzeng Xing**

Department of Urology, Beijing Chao‐Yang Hospital, Capital Medical University, Beijing, 100020, China

Department of Urology, National Cancer Center/National Clinical Research Center for Cancer/Cancer Hospital, Chinese Academy of Medical Sciences and Peking Union Medical College, Beijing, China

Correspondence to:

Nianzeng Xing, Department of Urology, National Cancer Center/National Clinical Research Center for Cancer/Cancer Hospital, Chinese Academy of Medical Sciences and Peking Union Medical College, Beijing, 100021, China.

Tel: +010 67781331

E‐mail: nianzeng168@163.com
