# Supplementary material for: LMTK3 promotes tumorigenesis in bladder cancer via the ERK/MAPK pathway
Source: FEBS Open Bio. 2020 Sep 16;10(10):2107–21. doi: 10.1002/2211-5463.12964 (PMC7530379; doi:10.1002/2211-5463.12964)
Supplement: Supplementary file 1 — Table S1. List of antibodies. [file FEB4-10-2107-s001.docx]

**Supplementary Table S1. List of antibodies.**

| **Antigens** | **Species antibodies raised in** | **Dilution (WB)** | **Supplier** |
| --- | --- | --- | --- |
| LMTK3 | Rabbit, polyclonal | 1:1,000 | Abcam, UK, Cat. #ab110516 |
| GAPDH | Mouse, monoclonal | 1:2,000 | Santa Cruz Biotechnology Inc., USA, Cat. #sc-365062 |
| p44/42 MAPK (Erk1/2) | Rabbit, monoclonal | 1:1,000 | Cell Signaling Technology, USA, Cat. # 4695 |
| Phospho-p44/42 MAPK (Erk1/2) (Thr202/Tyr204) | Rabbit, monoclonal | 1:1,000 | Cell Signaling Technology, USA, Cat. # 4370 |
| Cyclin B1 | Rabbit, monoclonal | 1:1,000 | Abcam, UK, Cat. #ab32053 |
| cdc25c | Rabbit, monoclonal | 1:1,000 | Abcam, UK, Cat. #32444 |
| Phospho-cdc25c | Rabbit, monoclonal | 1:1,000 | Abcam, UK, Cat. #47322 |
| cdc2 | Rabbit, monoclonal | 1:1,000 | Cell Signaling Technology, USA, Cat. #77055 |
| Phospho-cdc2 | Rabbit, monoclonal | 1:1,000 | Cell Signaling Technology, USA, Cat. #4539 |
| MEK1/2 | Rabbit, monoclonal | 1:1,000 | Cell Signaling Technology, USA, Cat. # 8727 |
| Caspase-3 | Rabbit, monoclonal | 1:1,000 | Cell Signaling Technology, USA, Cat. #9662 |
| Cleaved Caspase-3 | Rabbit, monoclonal | 1:1,000 | Cell Signaling Technology, USA, Cat. #9579 |
| Caspase-9 | Mouse, monoclonal | 1:1,000 | Cell Signaling Technology, USA, Cat. #9504 |
| Cleaved Caspase-9 | Mouse, monoclonal | 1:1,000 | Cell Signaling Technology, USA, Cat. #9509 |
| Phospho-MEK1/2 (Ser217/221) | Rabbit, monoclonal | 1:1,000 | Cell Signaling Technology, USA, Cat. # 3958 |
| E-cadherin | Rabbit, monoclonal | 1:500 | Cell Signaling Technology, USA, Cat. #3195 |
| N-cadherin, human | Rabbit, monoclonal | 1:500 | Cell Signaling Technology, USA, Cat. #13116 |
| β-Catenin | Rabbit, monoclonal | 1:1,000 | Cell Signaling Technology, USA, Cat. #8480 |
| Slug | Rabbit, monoclonal | 1:500 | Cell Signaling Technology, USA, Cat. # 9585 |
| Anti-Mouse-IgG (H+L)-HRP | Goat | 1:10,000 | Sungene Biotech, China, Cat. #LK2003 |
| Anti-Rabbit-IgG (H+L)-HRP | Goat | 1:10,000 | Sungene Biotech, China, Cat. #LK2001 |
